# Intensity of arterial structure acquired by Silent MRA estimates cerebral blood flow

**DOI:** 10.1186/s13244-021-01132-0

**Published:** 2021-12-11

**Authors:** Zhen-An Hwang, Chia-Wei Li, Chien-Yuan Eddy Lin, Jyh-Horng Chen, Chia-Yuen Chen, Wing P. Chan

**Affiliations:** 1 Department of Radiology, Wan Fang Hospital, Taipei Medical University, 111 Hsing Long Road, Section 3, Taipei 116, Taiwan; 2 Department of Radiology, School of Medicine, College of Medicine, Taipei Medical University, Taipei, Taiwan; 3 GE Healthcare, Taipei, Taiwan; 4 Department of Electrical Engineering, National Taiwan University, Taipei, Taiwan; 5 Neurobiology and Cognitive Science Center, National Taiwan University, Taipei, Taiwan

**Keywords:** Blood flow, Brain, Magnetic resonance angiography (MRA), Magnetic resonance imaging (MRI)

## Abstract

**Background:**

Cerebral blood flow (CBF) and the morphology of the cerebral arteries are important for characterizing cerebrovascular disease. Silent magnetic resonance angiography (Silent MRA) is a MRA technique focusing on arterial structural delineation. This study was conducted to investigate the correlation between Silent MRA and CBF quantification, which has not yet been reported.

**Methods:**

Both the Silent MRA and time-of-flight magnetic resonance angiography scans were applied in seventeen healthy participants to acquire the arterial structure and to find arterial intensities. Phase-contrast MRA (PC-MRA) was then used to perform the quantitative CBF measurement of 13 cerebral arteries. Due to different dataset baseline signal level of Silent MRA, the signal intensities of the selected 13 cerebral arteries were normalized to the selected ROIs of bilateral internal carotid arteries. The normalized signal intensities were used to determine the relationship between Silent MRA and CBF.

**Results:**

The image intensity distribution of arterial regions generated by Silent MRA showed similar laminar shape as the phase distribution by PC-MRA (correlation coefficient > 0.62). Moreover, in both the results of individual and group-leveled analysis, the intensity value of arterial regions by Silent MRA showed positively correlation with the CBF by PC-MRA. The coefficient of determination (*R*^2^) of individual trends ranged from 0.242 to 0.956, and the *R*^2^ of group-leveled result was 0.550.

**Conclusions:**

This study demonstrates that Silent MRA provides valuable CBF information despite arterial structure, rendering it a potential tool for screening for cerebrovascular disease.

**Supplementary Information:**

The online version contains supplementary material available at 10.1186/s13244-021-01132-0.

## Keypoints

- Imaging assessment of intracranial arteries and cerebral blood flow is essential in cerebrovascular diagnosis.
- Conventional TOF-MRA for evaluating intracranial arteries still faces some limitations and artifacts.
- Compared with TOF-MRA, Silent MRA provides additional cerebral blood flow information despite arterial structure

## Background

Evaluating cerebral hemodynamics, including vessel morphology and flow information, is important for characterizing the pathological features of acute stroke, chronic cerebrovascular disease, epilepsy, and brain tumors. In particular, morphological assessments of intracranial arteries are essential in cerebrovascular disease diagnosis. Therefore, imaging techniques focusing on intracranial artery delineation and cerebral blood flow (CBF) have been developed and applied clinically [1].

Conventionally, three-dimensional time-of-flight magnetic resonance angiography (3D TOF-MRA) has been used for evaluating morphology of intracranial arteries [2]. This contrast-agent-free technique uses the flow of spins to generate vessel contrast; it is sometimes considered as a follow-up imaging alternative to digital subtraction angiography [3]. Unfortunately, spin saturation effects (in slow flow) and phase dispersion artifacts (in turbulent flow) decrease signal intensity in 3D TOF-MRA [4–6], leading to overestimation of the severity of intracranial artery disease. Additionally, 3D TOF-MRA is prone to magnetic susceptibility and radiofrequency shielding artifacts. In addition, cerebral flow rate cannot be determined using 3D TOF-MRA.

As a result, phase-contrast quantitative magnetic resonance angiography (PC-MRA) without contrast agent has been developed to measure CBF as a volume flow rate (ml/min) [7, 8]. Zarrinkoob et al*.* [9] reported the distribution of total cerebral arterial flow across variations in age, sex, and anatomy using high-resolution PC-MRA establishing a normative reference value for blood flow in major cerebral arteries in healthy people [7]. Although PC-MRA can provide multidirectional flow and collateral flow, it does not improve stenosis detection in major intracranial vessels compared to 3D TOF-MRA because the optimized velocity-encoding gradient affects predominantly small vessels and the reduced number of partitions [10].

Silent magnetic resonance angiography (Silent MRA) is another MRA imaging technique [11–13]. As with 3D TOF-MRA, Silent MRA does not require contrast agent; it is a non-invasive perfusion imaging technique that uses continuous arterial spin labeling with a long radiofrequency inversion pulse, and it labels blood within the carotid arteries as an endogenous tracer [14, 15]. Subtracted by another image dataset without the use of arterial spin labeling, Silent MRA can depict the intracranial arteries without background tissues. However, with arterial spin labeling technique, Silent MRA tends to have drop-off on inflow enhancement [16]. Previous study reported that Silent MRA showed low signal intensity in distal vessels because of poor inflow (two of 27 intracranial Silent Scans) [17]. On the other hand, it implied that the signal intensity obtained by Silent MRA contained not only the structure of arteries but also the flow information.

Moreover, with the use of a zero echo time technique, Silent MRA is able to minimize the phase dispersion of the labeled blood flow signal and decrease magnetic susceptibility compared to 3D TOF-MRA [18]. Its use has recently been proposed for assessing vascular lesions such as those in treated intracranial aneurysms [18–21] and in cerebral arteriovenous malformations [22, 23]. These studies show that it provides excellent architectural visualization in coiled aneurysms and flow through intracranial stents.

Studies have focused primarily on delineating vascular lesion structures using a combined zero-echo-time and arterial spin labeling technique, but none have thoroughly investigated the use of Silent MRA for estimating CBF. Therefore, we aimed to explore the usefulness of Silent MRA for flow estimation. We studied the correlation between Silent MRA and CBF as estimated using PC-MRA in a healthy population.

## Methods

### Participants

Seventeen healthy volunteers (8 women; mean age, 33.8 ± 7.1 years) joined this study between October 2018 and July 2019. None had claustrophobia, psychological disorders, cardiac pacemakers, contraindications to magnetic resonance imaging, or metal implants, and none were pregnant. During the entire scanning session, the participants were to maintain a motionless head. Participants remained awake to prevent unwanted motion artifacts. This study was approved by the Research Ethics Committee of Taipei Medical University—Joint Institutional Review Board (N201803017), and informed consent was obtained from all participants.

### MRA scanning

All magnetic resonance images were acquired using a 3.0-T clinical scanner (Discovery MR750w; GE Healthcare, Milwaukee, USA) equipped with a 24-channel Geometry Embracing Method head-and-neck coil for signal detection and a whole-body coil for radio-frequency excitation. Both the Silent MRA and 3D TOF-MRA scans were used to acquire the arterial structure before performing 2D PC-MRA. The parameters for acquiring the Silent MRA were: repetition time, 759.924 ms; echo time, 0.04 ms; flip angle, 5°; field of view, 20 cm; matrix, 256 × 256; section thickness, 1.4 mm; NEX, 1.0; bandwidth, 62.5 kHz; and acquisition time, 5 min 15 s. The parameters for acquiring the 3D TOF-MRA were: repetition time/echo time, 19 ms/2.9 ms; flip angle, 15°; field of view, 18 cm; matrix, 416 × 192; section thickness, 1.2 mm; NEX, 1; bandwidth, 41.7 kHz; and acquisition time, 3 min 31 s.

In the 2D PC-MRA flow-measuring scan, a Non-Invasive Optimal Vessel system (NOVA; VasSol, Inc., Chicago, IL, USA) was used to perform the quantitative flow measurements in 13 cerebral arteries: the basilar artery, the bilateral anterior cerebral arteries, the superior branches of the bilateral anterior cerebral arteries, the bilateral middle cerebral arteries, the bilateral posterior cerebral arteries, the bilateral vertebral arteries, and the bilateral internal carotid arteries. The parameters for acquiring the PC MRA were: repetition time/echo time, 12.82 ms/5.728 ms; flip angle, 25°; field of view, 16 cm; matrix, 256 × 256; section thickness, 5 mm; NEX, 1; bandwidth, about 162.734 kHz; VEC, automatically selected by NOVA software. To minimize the effects of turbulence, flow was measured at the point furthest from vessel turns within the designated segment. Perpendicular imaging for any given vessel was automatically created by the NOVA system so that arterial flow could be accurately measured. A laminar flow model was used to calculate vascular flow rates. The vessels used to designate flow status were those in distal territories of the vertebrobasilar tree. Quantitative flow was then measured in the chosen vessels via the 2D PC-MRA technique using the NOVA system. Data were acquired under the guidance of one experienced (25 years) radiological technologist.

### Silent MRA data analysis

**Fig. 1:**
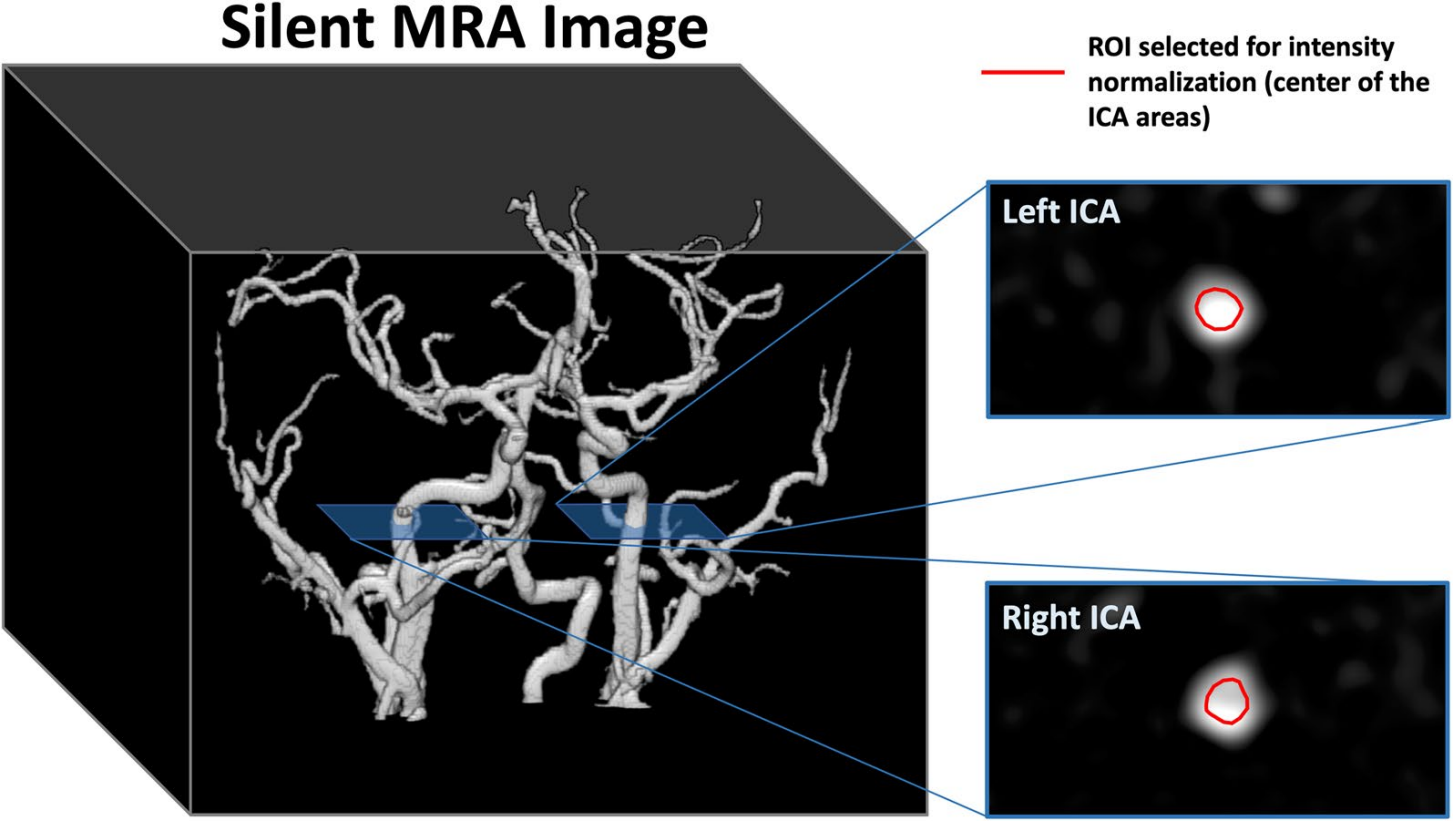
Locations of selected ROIs (marked with red lines) based on bilateral cervical segments of internal carotid arteries (ICA) for intensity normalization. The magnitudes of the individual Silent MRA data were divided by the average intensity value of the two selected ROIs for the following group-level analysis

We compared the arterial structure and vessel uniformity of bilateral internal carotid arteries (ICAs) between Silent MRA and 3D TOF-MRA. To interpret the flow information obtained using Silent MRA, average image intensities based on the exact location of the 13 cerebral arteries in PC imaging were calculated, and the flow-encoding Silent MRA signal distribution was compared with that obtained using PC flow imaging. Because the baseline of each individual Silent MRA dataset resulted in a different signal level, the image intensities of the 13 arterial areas were normalized to the central regions of the selected ROIs located prior to the bilateral cervical segments of ICAs (Fig. 1). In individual analyses, the normalized intensities of the arterial regions, generated using Silent MRA, were correlated to the corresponding flow rates generated using PC imaging. In the group-level analysis, one linear regression was applied across all the normalized arterial intensities and their corresponding flow rates, allowing investigation of the relationship between the normalized arterial intensities and CBF. To explore the relationship between the normalized arterial intensity from the Silent MRA and the quantified CBF, linear regression with 95% confidence intervals was calculated. Statistical analyses were performed using R version 3.5.1 (R Core Team 2018. R: A language and environment for statistical computing. R Foundation for Statistical Computing, Vienna, Austria. URL http://www.R-project.org/).

## Results

**Fig. 2:**
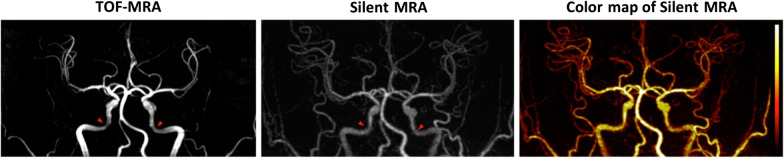
Arterial structures acquired using Silent MRA and 3D TOF-MRA. Red arrows indicate the petrous segments of internal carotid arteries. Silent MRA shows comparatively less signal loss. Using the arterial spin-labeling technique, Silent MRA provides flow information in the gradient magnitudes of the arterial regions

**Fig. 3:**
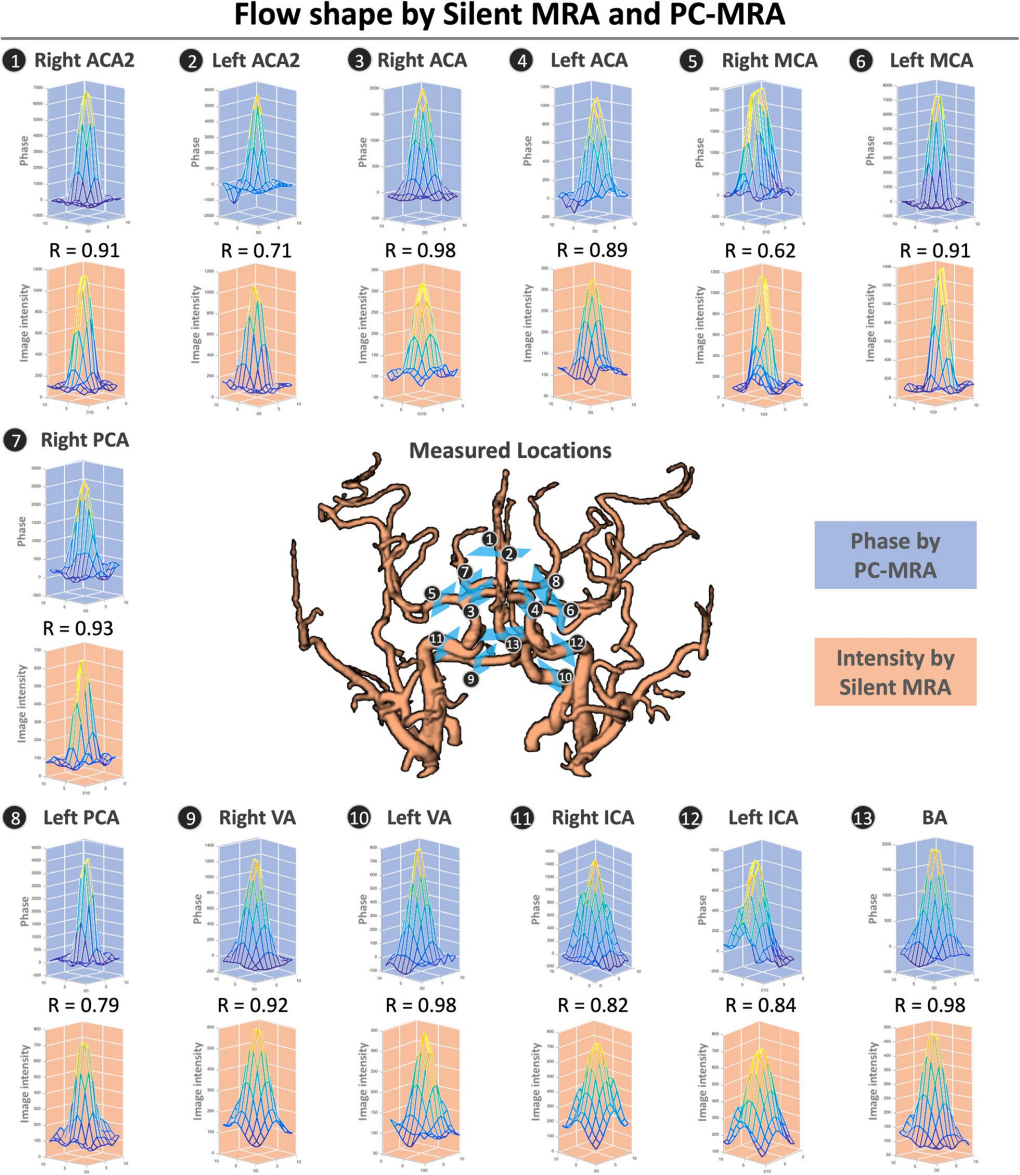
Comparisons of flow shapes generated by Silent MRA and 2D PC-MRA. The laminar flow shapes of the 13 cerebral arteries of one study participant (33-years-old; man) are similar whether generated by 2D PC-MRA or Silent MRA. The variable R is the correlation coefficient between the phase distribution (in 2D PC-MRA) and signal intensity (in Silent MRA)

Compared with 3D TOF-MRA, Silent MRA provided more detail in the arterial structure and a more uniform intensity in the petrous segments of internal carotid arteries (one example was listed in Fig. 2, and all the dataset were listed in the Additional file 1: Fig. S1), and it showed comparatively less signal loss in the Silent MRA. Additionally, its structural map showed gradient intensities that resulted from flow-weighted information, such as laminar-shaped flows, in the arterial regions. Figure 3 shows the demographic data of the laminar-shaped phase distribution via 2D PC-MRA and the intensity variations of the 13 cerebral arteries via Silent MRA. All phase distributions acquired using 2D PC-MRA yielded a laminar shape, and the intensity distribution shown by Silent MRA agreed with that shape (correlation coefficients of arterial flow-shapes by 2D PC-MRA and Silent MRA are all larger than 0.6). The intensity variation shown by Silent MRA highly correlated with the distribution of phase shown by 2D PC-MRA; their correlation coefficients ranged from 0.62 to 0.98.

**Fig. 4:**
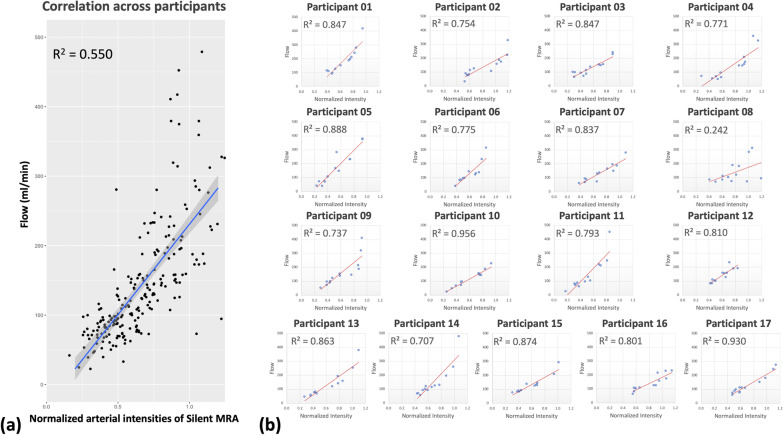
Linear correlations between the normalized arterial intensities in Silent MRA and CBF determined using 2D PC-MRA. **a** Correlation between the normalized arterial intensities and the corresponding CBFs was highly linear (*R*^2^ = 0.550). The dark grey area indicates the 95% confidence interval of the linearly fitted line. **b** At the individual participant level, normalized arterial intensity was also highly linearly correlated

Table 1 compares the CBF values generated using 2D PC-MRA for each of the 13 arteries to the image intensities generated using Silent MRA at the same arterial location. The normalized arterial intensity was adopted at each arterial location in the group-level analysis, and a linear relationship (*R*^2^ = 0.550) was found (Fig. 4). For every participant, the normalized arterial intensities across the 13 arterial regions were highly positively correlated with the corresponding CBFs: *R*^2^ ranged from 0.242 to 0.956. Linear regression was also used to determine the relationship between the normalized arterial intensities in the Silent MRAs and the quantified CBFs. With 95% confidence, the group-level result predicts the CBF range when using the following:

1 $${\text{CBF}}\;{\text{ range}} \left( {{\text{within}}\; 95\% \;{\text{ confidence}}\;{\text{interval}}} \right) = \left\{ {\begin{array}{*{20}c} {\left( {{\text{upper}}\;{\text{value}}} \right) 292.1 - 7.479 \times {\text{SNR}}_{{\text{silent-MRA}}} } \\ {\left( {{\text{lower}}\;{\text{value}}} \right) 228.9 - 51.67 \times {\text{SNR}}_{{\text{silent-MRA}}} } \\ \end{array} } \right.$$

**Table 1:** Image intensities in silent MRA and corresponding flow rates determined using 2D PC-MRA

Participant		R A C A 2	L A C A 2	R A C A	L A C A	R M C A	L M C A	R P C A	L P C A	R V A	L V A	R I C A	L I C A	B A
1	Silent MRA	759	755	832	964	1231	1308	622	665	–	1164	1509	1342	1200
	Flow rate	95	92	127	152	213	241	116	110	–	188	417	280	197
2	Silent MRA	566	585	599	659	1001	1157	533	560	521	1066	1168	1040	918
	Flow rate	76	83	114	124	158	223	90	78	33	173	328	188	107
3	Silent MRA	444	704	581	791	981	1020	410	455	642	697	1283	1285	1074
	Flow rate	66	87	93	138	153	150	100	98	73	115	229	242	157
4	Silent MRA	605	538	697	638	1122	1136	341	689	1023	1085	1281	1373	1113
	Flow rate	70	55	63	50	158	173	71	96	147	147	359	326	208
5	Silent MRA	495	321	740	489	719	1023	376	554	439	1029	1286	1288	789
	Flow rate	69	42	281	72	166	232	71	108	39	232	375	379	147
6	Silent MRA	705	578	884	–	1116	1045	689	674	784	752	1275	1180	1030
	Flow rate	85	40	144	–	139	131	85	83	98	97	314	233	123
7	Silent MRA	586	472	1113	–	862	1014	586	613	813	1128	1364	1195	817
	Flow rate	93	62	196	–	136	165	67	87	73	150	280	188	129
8	Silent MRA	554	667	453	675	950	842	1326	1087	824	767	1173	1114	901
	Flow rate	70	85	86	112	182	189	95	73	74	102	312	285	121
9	Silent MRA	370	552	591	738	1081	1095	483	545	730	487	1152	1136	953
	Flow rate	53	99	122	139	213	188	72	90	153	97	411	319	146
10	Silent MRA	523	444	334	991	1006	1040	632	648	1121	632	1016	1245	1007
	Flow rate	68	48	23	157	144	143	94	97	187	73	145	227	152
11	Silent MRA	457	448	726	532	962	998	498	439	246	774	1188	1132	659
	Flow rate	76	71	129	61	218	212	88	80	25	102	452	247	96
12	Silent MRA	663	741	905	652	948	1059	672	627	971	721	1005	1249	1146
	Flow rate	84	100	158	83	155	233	109	83	128	102	159	192	190
13	Silent MRA	469	585	540	556	885	1002	468	559	1089	349	1285	1401	991
	Flow rate	59	68	76	78	119	141	52	80	158	46	253	379	194
14	Silent MRA	532	651	1035	777	917	829	516	617	702	568	1261	1164	671
	Flow rate	70	100	195	114	130	126	68	92	92	56	479	259	121
15	Silent MRA	529	394	669	563	879	865	491	489	828	514	1202	1296	875
	Flow rate	82	77	138	92	144	131	82	83	124	90	209	294	130
16	Silent MRA	673	648	672	774	1099	1238	700	702	1064	1024	1251	1353	1124
	Flow rate	81	64	108	110	215	228	102	106	126	125	174	231	159
17	Silent MRA	624	613	617	506	938	1036	538	559	506	655	1202	1162	707
	Flow rate	79	76	112	59	152	180	88	98	74	111	276	245	109

The RVA data from participant 01 and the LACA data from participants 06 and 07 were excluded from this study due to excessive motion artifacts

*Int.* Intensity; The unit of flow rate: ml/minutes

## Discussion

The novel contributions of this study are its investigation into the flow-weighted image intensity of Silent MRA, and the determination of a relatively straightforward linear relationship that flexibly connects the normalized arterial intensities in Silent MRA with quantified CBF. Expectedly, Silent MRA provides more details in the arterial structure and a more uniform intensity in the petrous segments of the internal carotid arteries compared to 3D TOF-MRA. Fujiwara et al. [24] similarly showed it was superior to 3D TOF-MRA in carotid artery uniformity and blood vessel contrast. Our results show that with the advantages of arterial spin labeling and zero echo time, Silent MRA provides image intensity distributions that are highly correlated with the phase distributions generated by 2D PC-MRA in these arterial sections. The two demonstrate similar laminar shapes in the studied vessel sections. Moreover, the normalized arterial intensities in Silent MRA are highly positively correlated with the flow rates provided by 2D PC-MRA at the individual participant level; at the group level, results are highly linear as well. Using linear regression and the 95% confidence interval, the normalized arterial intensity provided by Silent MRA can be indirectly used to predict the corresponding CBF range in these arterial regions. For example, a normalized arterial intensity of 0.7 in the Silent MRA image corresponds, with 95% confidence, to a CBF ranging from 192.1 to 286.9 ml/min. Therefore, one 5-min Silent MRA can provide both the structural information and a relative quantification of CBF from a 2D PC-MRA, implying its potential role in screening for arterial disease.

Studies examining the relationship between Silent MRA and 2D PC-MRA have not yet been discussed in the literature, and our study shows that Silent MRA can provide more flow information than 3D TOF-MRA. Conventionally, a low intraluminal signal intensity on a 3D TOF-MRA could be the result of a slow flow state such as a stenosis or occlusion, but this modality reportedly overestimates the degree of intracranial artery stenosis [25]. Additionally, phase dispersion artifacts from turbulent flow can result in diminished signal intensity in a 3D TOF-MRA [4–6]. With the use of zero echo time technique, Silent MRA reduces slow flow related signal dropout, rendering it more reliable than 3D TOF-MRA in evaluating slow flow status such as carotid stenosis. Acute phase of thrombus is reported to show high signal intensity in TOF-MRA especially in the cavernous segment of internal carotid arteries [18, 26, 27]. Silent MRA integrates continuous arterial spin labeling with the generation of angiographic images by subtracting labeled and nonlabeled blood flow images. Presumably the signal change attributable to acute thrombus formation can be subtracted, and static tissue such as thrombus cannot be detected by Silent MRA [18]. Oishi et al. [26] reported high rate of false-positive signals of intra-aneurysmal thrombosis on TOF-MRA and concluded that Silent MRA showed better depiction of intra-aneurysmal embolization status compared with TOF-MRA. An intracranial stenosis and occlusion attributable to a thrombus could be differentiated using this method. Therefore, Silent MRA can be considered a screening tool for detecting intracranial steno-occlusive disease. Another concerning issue is that the diagnostic role of Silent MRA in chronic small vessel disease has not been well established. Chronic small vessel disease and lacunar infarcts usually involve penetrating arteries and arterioles of Willis’ circle. Previous studies showed that Silent MRA was superior to conventional TOF-MRA for visualizing Moyamoya vessels, which referred to the dilated perforating arteries [28, 29]. Therefore, the potential of Silent MRA in depicting penetrating arteries is worth to be studied.

Cerebral blood flow plays a crucial role in ischemic cerebrovascular disease, and asymmetry in CBF correlates with disease severity and clinical outcome [30]. As this study shows, the signal intensity generated by Silent MRA and an independent CBF measurement are highly positively correlated. The signal intensity seen on the Silent MRA can indirectly imply intraluminal CBF. We presume that a greater intraluminal vessel signal intensity reflects better blood flow in recanalized arteries; therefore, Silent MRA can be applied in acute ischemic stroke to observe arteries following thrombectomy or to determine outcomes following thrombolysis. Recent studies report promising results using Silent MRA as follow-up imaging when assessing coiled or stent-assisted coiled intracranial aneurysms [18–21]. Similarly, stent flow visualization can suggest increased intraluminal CBF despite patency.

Silent MRA has two primary clinical disadvantages. There is marked background suppression with loss of anatomical landmarks, particularly with normal variants of intracranial arteries. However, background suppression shows better blood vessel contrast. Baseline image studies such as computed tomography angiograms or digital subtraction angiographies are performed first and can be used as references, rendering Silent MRA useful as follow-up imaging for treated intracranial arteries. Secondly, compared to 3D TOF-MRA, the longer acquisition time could increase the possibility of motion artifacts.

## Limitation

This study has several limitations. First, it is a single-center cohort study with small number participants and fixed scanning parameters; however, it provides promising preliminary results not yet found in the literature. Second, it was limited to young healthy participants. Initially, this was part of the study design to avoid potential bias associated with disease states. Further prospective study with larger population is needed to validate the diagnostic value of Silent MRA in estimating cerebral blood flow in older participants with cardiovascular risk or intracranial stenosis. Third, Silent MRA is independent of flow direction. Therefore, cerebral hemodynamic changes with arterial steal and flow reversal, as with subclavian steal syndrome or reversed Robin Hood syndrome, might not be detected. This could be solved by inducing advanced arterial spin labeling such as vessel-selective labeling [31, 32]. Fourth, it is difficult to select a common standard from images by Silent MRA for normalization due to the absence of background tissues. The standard used for normalization in this study is the ROI intensity of bilateral ICAs. However, it also contains the flow information within the selected ROIs. This results in the vary correlation between the intensity of Silent MRA and the flow by 2D PC-MRA among participants, which responses to the lower group-leveled result (*R*^2^ = 0.550) while compared to individual results (most *R*^2^ > 0.7). It may be solved by a better standard, such as the non-spin-labeling images reconstructed from the individual raw k-space, used for normalization. Fifth, variation of the intracranial artery is not unusual. For example, some degree of asymmetry between the two A1 segments was noted in up to 80% of cases [33], and the frequency of hypoplastic vertebral artery has been reported in up to 26.5% of normal healthy population [34]. The distribution of the signal intensity and vessel contrast generated by Silent MRA may result in alterations. For the middle cerebral artery region, we only measured M1 segments bilaterally, and further labeling of the M4 segments should be evaluated in future study. Lastly, Silent MRA is currently not feasible for single artery measurement and does not provide assessment of its CBF quantification.

In conclusion, the normalized arterial intensity in Silent MRA and the corresponding CBF generated by 2D PC-MRA are positively correlated, and the former can be used to predict the range of the latter. By providing a better depiction of the arterial structure and additional CBF information compared to 3D TOF-MRA, Silent MRA is a potential tool for screening steno-occlusive disease and providing supporting imaging information.

## Supplementary Information

**Additional file 1**. **Figure S1**. Maximum intensity projection (MIP) images from TOF-MRA and Silent MRA. Compared with TOF-MRA, Silent MRA provides more uniform signal intensity of bilateral ICAs with less signal loss (yellow arrowheads).

## Data Availability

The datasets generated and analyzed during the current study are not publicly available due to the policy of the Research Ethics Committee of Taipei Medical University—Joint Institutional Review Board but are available from the corresponding author on reasonable request.

## Abbreviations

ASL: Arterial spin labeling
CBF: Cerebral blood flow
MRA: Magnetic resonance angiography
PC: Phase-contrast
SNR: Signal-to-noise ratio
TE: Echo time
TI: Inversion time
TOF: Time-of-flight
TR: Repetition time
