# Supplementary material for: Intensity of arterial structure acquired by Silent MRA estimates cerebral blood flow
Source: Insights Imaging. 2021 Dec 11;12:185. doi: 10.1186/s13244-021-01132-0 (PMC8665965; doi:10.1186/s13244-021-01132-0)
Supplement: Supplementary file 1 — Additional file 1. Figure S1. Maximum intensity projection (MIP) images from TOF-MRA and Silent MRA. Compared with TOF-MRA, Silent MRA provides more uniform signal intensity of bilateral ICAs with less signal loss (yellow arrowheads). [file 13244_2021_1132_MOESM1_ESM.docx]

**ELECTRONIC SUPPLEMENTARY MATERIAL**


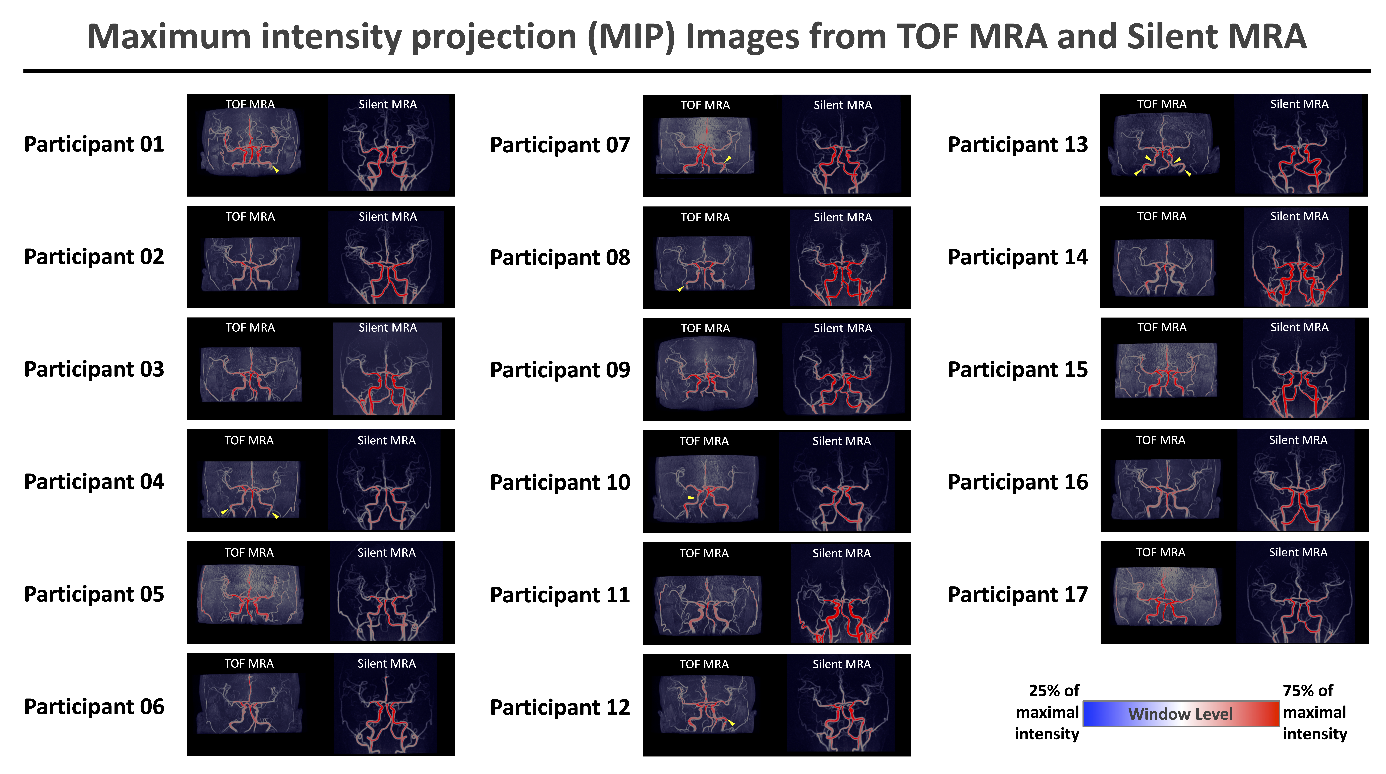
 **Figure S1. Maximum intensity projection (MIP) images from TOF-MRA and Silent MRA.** Compared with TOF-MRA, Silent MRA provides more uniform signal intensity of bilateral ICAs with less signal loss (yellow arrowheads).
